# Emotional contagion and proto-organizing in human interaction dynamics

**DOI:** 10.3389/fpsyg.2015.00806

**Published:** 2015-06-12

**Authors:** James K. Hazy, Richard E. Boyatzis

**Affiliations:** ^1^Adelphi University, New York, NY, USA; ^2^Mälardalen University, Västerås, Sweden; ^3^Case Western Reserve University, Cleveland, OH, USA

**Keywords:** complexity, group dynamics, social contagion, emotional states

## Abstract

This paper combines the complexity notions of phase transitions and tipping points with recent advances in cognitive neuroscience to propose a general theory of human proto-organizing. It takes as a premise that a necessary prerequisite for organizing, or “proto-organizing,” occurs through emotional contagion in subpopulations of human interaction dynamics in complex ecosystems. Emotional contagion is posited to engender emotional understanding and identification with others, a social process that acts as a mechanism that enables (or precludes) cooperative responses to opportunities and risks. Propositions are offered and further research is suggested.

## Introduction

There has been an increasing call for approaches that might be used to build theoretical microfoundations for social theory (Devinney, 2013; Greve, 2013; Winter, 2013). When complexity of organizing is addressed, it is often treated as a product of rational choice and individual decisions about whether to cooperate and how to organize (von Neumann and Morgenstern, 1944; Axelrod, 1984, 1997). Increasingly, the role of rational choice has been questioned (Kahneman, 2011). Still, even these studies tend to focus on the neural mechanisms that underlie reasoning processes, whether analytic or intuitive processes are activated, rather than more primal emotional ones. Even business school cases and situation reports often imply that the process is indeed guided by rational choice.

This article contributes a new way of thinking by taking a contrarian approach. We argue that the drive to cooperate within human populations may have preceded, in an evolutionary biology sense, the complex rational cognitive processing that are assumed to enact decision making. In the proposed model, we suggest that the dynamics of human organizing might be a primordial aspect of human society that derives from emotional cognitive systems and a phenomenon known as *emotional contagion* rather than from rational decision-making processes. Individuals in groups experience rapid, localized feedback about the emotional states of others. This influences their own emotional states, which influences the emotions of others, and so on, creating a collective, synchronized emotional state among a subset of individuals within the population (Boyatzis et al., 2015).

This emotional contagion may lead to a shared community-identity about how one’s feelings about the situation (rather than what one thinks about it) might impact the choices and actions that might need to be taken. Because everyone feels the same way, each individual can better anticipate how others will respond to events. Their “action vectors,” as one might call them, can be observed to align toward a common direction of action. Under these conditions, the subpopulation is primed to act “as one.” We call this process “proto-organizing” to distinguish it from rational organizing that is orchestrated under a clearly articulated collective purpose. When proto-organizing remains stable over time, even when some individuals leave and others join the population, we call this stable dynamic pattern “proto-community.”

In the first section of this article, we contend that individuals tend to be in one or the other of two distinct emotional states: a positive emotional attractor (PEA; Howard, 2006; Boyatzis, 2008; Boyatzis et al., 2015) or a negative emotional attractor (NEA). In the second section, we contend that in uniform, sparsely connected, unconstrained populations, individuals experience one or the other of these states based upon individual traits and locally relevant conditions (e.g., the quality of their close relationships). The distribution of emotional states in a large, unconstrained population in a benign environment can be approximated for modeling purposes using random distribution assumptions. When the emotional states of many in the population are sampled, observed and measured, the central tendency values for the population (i.e., the mean) might vary according to global conditions such as seasonality or the availability of resources. However, these values would be consistent across samples as local effects would tend to cancel each other out in a large population.

In the third section, we contend that when certain parametric conditions develop in the ecosystem (e.g., a newly perceived opportunity or risk) one of two states can become “contagious” (Goldstein et al., 2010a,b; Hazy and Ashley, 2011). As the infection spreads, the population moves into a dominant emotional state. In the fourth section, the Dodds and Watts (2004) general model of social contagion is described. In the fifth section, the above elements are combined into the beginning of a theory of proto-organizing as a set of phase transitions in emotional states within populations.

## The Bifurcated Emotional States of Individuals

The theory we propose consists of multiple elements. The first of these is that individuals can be modeled as heterogeneous *agents* that have both emotional and rational cognitive processes. For this study, we focus on the relevance of emotional states.

Boyatzis (2008) and Boyatzis et al. (2015) described each human as (almost) always being in one of two distinct emotional states as a consequence of neurological and hormonal functioning. The NEA is a state that is defined by the individual, dyad, team or larger human system as aroused in the sympathetic nervous system (SNS), feeling negative affect and activating the neural task positive network (TPN), the higher the NEA state (Boyatzis et al., 2015). This state puts individuals—and thus families and teams who share it—into a defensive mode, prone to caution, risk aversion, and analysis. The body moves to protect itself which, if prolonged, can result in the brain going into cognitive, perceptual, and emotional impairment (Boyatzis et al., 2006). The SNS is more commonly known as the stress response (Sapolsky, 2004). The TPN is a neural network within the executive function that is useful in focusing attention, solving problems and analysis (Jack et al., 2012). As Jack et al. (2012) showed, when the TPN is activated another neural network associated with people, emotions and being open to new ideas is suppressed.

In contrast, PEA is a state that is defined by the human or human system aroused in the parasympathetic nervous system (PNS), feeling positive affect, and activation of the default mode network (DMN; Boyatzis et al., 2015). This state puts individuals—and thus families and teams who share it—into an open and social mode, prone to scanning the environment, seeing others and their feelings and perceiving moral concerns (not moralizing). The PNS is considered the body’s renewal system. It allows a human to rebuild oneself and recover from effects of the SNS (Boyatzis et al., 2006). The DMN is a neural network that is useful in being open, scanning the environment, including the social environment, being creative, handling more complex cognitive tasks and multi-tasking (Jack et al., 2012). Jack et al. (2012) showed that when the DMN is activated, neural networks like the TPN which are associated with focusing, problem solving and analysis are suppressed. These effects work the same for the functioning of the immune system—in the NEA the immune system is compromised, and in the PEA it is healthy.

In complexity terms, Boyatzis (2008) and Boyatzis et al. (2015) argue that these two states are each likely to be manifestations of neurological dynamical states that are constrained within strange attractors (Lorenz, 1963). These attractors are arrayed on three dimensions: (1) positive to negative affect; (2) parasympathetic to sympathetic nervous system activation; and (3) activation of the neural networks TPN or DMN. Each emotional state is self-sustaining within a given individual and remains relatively stable. Even when subject to relatively mild perturbations or to relevant events, the individual’s emotional state may change to a degree from the event, but eventually settle backs into its prior emotional state, either PEA or NEA. This would remain true until a tipping point within an individual’s internal state is reached, perhaps precipitated by a significant event or “trigger.” A trigger might be a negative event like feeling frustrated with one’s computer’s slow speed, having someone cut one off in traffic, or becoming angry with one’s spouse. A tipping point could also be a positive event like feeling grateful to someone, laughing, feeling a burst of hope, or feeling one with others or with the environment.

Tipping points act like the peak of a mountain ridge separating two adjoining valleys one to retain each stable condition, NEA or PEA as a personal disposition. If a perturbation, whether strong or mild, exceeds a “threshold” value, it can push someone who had been in a PEA state beyond the ridge or “tipping point” that separates the two attractors. The neurological, hormonal, and affective system can “flip” to the other state transitioning the individual into an NEA state. Continuing the mountain ridge metaphor, one’s emotional state “falls” into the “NEA attractor valley” beyond the ridge separating the two states.

The tipping points moving from one attractor within the neurological dynamical states inside the individual to another—either PEA → NEA, or NEA → PEA—is assumed to occur close to the 0 point of these three axes (Boyatzis et al., 2015). For example, when in the midst of a shouting argument with your spouse or partner is not the moment to try to “tip” them into the PEA by telling them to calm down. It often does the opposite and further intensifies their negative affect sending them further into the NEA. But if you made a pot of coffee, sat on a bench outside, asked your partner or spouse to join you, and then enjoyed the setting for a few moments of silence, the intensity of the affect might be reduced. Unless they are still seething, both people might have also decreased the surge of hormones from the NEA/SNS and even enjoyed just a peaceful moment. That might bring you both close to a tipping point—or not!

Negative events (over the threshold) for individuals can trigger their defensive, analytical NEA state. Threats must be analyzed; risk mitigated. This state may also strengthen their tendency to rely on basic natural categories (Rosch et al., 1976) to organize perception in their cognitive processes as a means to simplify their responses. In this way, human beings need the NEA to *survive* stress in the ecosystem (Boyatzis et al., 2015). For example, selection pressures in the ecosystem can make periodic culling of the population severely felt leading to a sense of personal isolation and risk. This would create conditions wherein trusting relationships are less likely to be sustained. Individuals must be rational, careful, and calculating to survive. One would expect the choice to cooperate to be equally rational and tinged with self-interest.

In contrast, a positive event (over a threshold) can trigger the PEA state which promotes openness and willingness to explore possibilities, where the individual relies more on intuition and confidence. This state may lessen one’s tendency to rely on basic natural categories (Rosch et al., 1976) when deciding how to respond and thus leave open the possibilities of social interaction and learning as well as the willingness to cooperate with others. Humans can rely on the PEA to thrive when conditions in the ecosystem allow that the act of “trusting others” is perceived to carry little risk. This would be the case when the population has had little turnover allowing trusted and efficacious relationships to be nurtured and sustained over many interactions and in multiple projects (Boyatzis et al., 2015). Under these conditions, one would expect the choice to cooperate to be almost assumed, the default strategy, to be perceived as a “given.”

In a later section we describe how collective emotional states might be described through models of social contagion. For example, one would expect that when NEA becomes dominant, individuals experience stresses from their ecosystem together as a group. When individuals survive, they do so together. If in the process, they build trusting relationships which they can count on for useful information including emotional information, the relationships themselves may become a basis for a tipping point into the PEA as they share and help each other. The experience of gratitude and compassion are key emotions that can tip a person into the PEA.

In dense social environments (beyond a certain threshold density level), either of these emotional states can quickly be transferred from some individuals to others through interactions and the feedback effects of emotional and social contagion, the process of transition from “me” to “we” that is discussed in a later section. Before discussing this, however, we first describe the baseline assumptions that we posit as a foundation for a formal model.

## Distribution of Emotional States within Populations

Absent exogenous constraining forces or events in the ecosystem and assuming a resource rich environment with little predation or competition, a population of individuals within the ecosystem might be assumed to form a sparsely connected population of largely autonomous individuals or affinity groups. Each of these individuals would adopt one or the other of the two emotional states based upon internal physiological and neurological dynamics as influenced by individual traits, locally relevant conditions, and the quality of their relationships. For example, family members might influence one another, but when viewed from the perspective of a large enough population, these localized effects would tend to cancel each other out. Ignoring these local effects, one might measure the aggregate emotional state at the population level with a macroeconomic indicator such as the Conference Board’s consumer confidence index^®^, or on the negative side, as a measure of escalating obesity, average blood pressure, or feelings of anxiety or distress.

In this simple baseline case that we call the *substrate*, we assume that each individual’s emotional state is individually, independently, and identically distributed and drawn from a uniform distribution of individuals with either an NEA or a PEA state, albeit their relative concentrations might vary depending upon the current macro-conditions in the ecosystem. In a large population acting as the substrate, however, we initially assume a random distribution wherein these local effects cancel one another out in the aggregate and therefore can be ignored.

The emotional state of others is locally observable (and increasingly so with social media), however, and these observations contain information to be recognized and gathered by others. As a result, in the more general cases discussed in later sections, information gathered about the emotional states of others might be useful to individuals—among animals, a deer might flash the white underside of its tail as a warning, for example, or a beaver might slap the water with its tail. Information about the emotional state of others thus becomes an additional factor influencing the emotional state of any particular individual. When synchronized patterns associated with the flow or *transit* of emotional state information emerge across the population, something else may be happening. These emergent patterns might signal a “phase transition” which occurs when the way that a system is organized suddenly changes and the system becomes organized in a new way. In a later section we describe a formal model that describes the phase transition in a human population from one that is the randomly distributed substrate to one that is either predominantly PEA or NEA. First, however, we further explore the substrate of human populations upon which emotion contagion might operate.

### Autonomous Agents Imply a Uniform Distribution

To begin, we define the substrate for this process by taking the simplifying assumption and treat PEA and NEA attractor states in individuals as representing two fixed point attractor states that characterize the two possible states for each individual within the population. This means that in the population model, individuals are treated as being in either the PEA state or the NEA state, regardless of the internal dynamics and neurological complexity within each individual’s brain. For simplicity, transitional states and the subjective experience of changing ones emotional position are treated as noise from the population perspective.

Each individual is assumed to be in or shift between two dynamically stable emotional states as individuals go through internal transitions in emotional state (a process that is beyond the scope of this paper). For a population of N individuals, the state, *γ*_i_, of individual *i* = [1, N], is *γ*_i_ = 1 if the state is PEA, and it is *γ*_i_ = –1 if individual *i* ϵ *N* is in the state NEA. Assuming each individual is equally predisposed with a given probability *p* ϵ [0, 1] to be either PEA or with probability 1–*p* to be NEA, and assuming each individual adopts its emotional state independently of its neighbors, the state of the system of N individuals, denoted *φ(N)* is a random variable with a uniform distribution where each draw (i.e., an individual’s momentary state) has the probability *p* to be 1 and *1–p* for it to be –1.

For illustrative simplicity, we assume that the mean for the population is normalized to 0—that is, *p* = 0.5, so that a randomly chosen individual’s state is equally likely to be PEA or NEA. The consumer confidence index^®^ mentioned earlier and normalized in this way might be a proxy for this parameter. In practice, empirical studies might find that the mean of the substrate population may vary over time and lean either toward NEA or PEA in which case, the probability *p* would not be 0.5 and the mean emotional state for the population would not be 0. Because the value of the parameter can be normalized, however, this simplifying assumption does not materially impact the generalizability of this analysis.

### Interacting Agents Imply Correlated Emotional States

As population density increases and individuals begin to interact in close-quarters, it becomes necessary to relax the assumption that an individual’s emotional state is independent of his or her neighbors. Research in social psychology has shown that emotional attractor states can be contagious and transmitted from one individual to another, spreading like an infection and thus potentially modeled using epidemiology models (Hatfield et al., 1993; Fowler and Christakis, 2008, 2010). Research in neuroscience has shown that the role of mirror neuron networks (Iacoboni, 2009) and sympathetic hemodynamic networks (Decety and Batson, 2007) enable a person to unconsciously and quickly (within milliseconds) tune into the actions of others and their feelings. Emotional contagion spreads quickly and unconsciously to others because of the work of the von economo neurons (Allman et al., 2005).

Emotional and social contagion depends, of course, not only on the ability of other individuals to correctly recognize the signal of an emotional state through body positioning, facial expressions, or verbal cues (cf. Aviezer et al., 2012), but also unconscious sympathetic hemodynamic processes in which brains affect other brains around them (Decety and Batson, 2007). The reader should note that for this analysis, the non-rational, non-symbolic transmission of emotional states among individuals is treated as an aspect of *information transit* within populations. This is a point of departure from many theoretical analyses which equate rational thought with information processing and treat emotions as something else entirely or choose to ignore the relevance of emotional life in organizations altogether.

More precisely, in cases of population density above a certain threshold, this amended assumption with regards to interaction affects can be stated as follows: under certain conditions, interactions between individuals can trigger phase transitions in interacting pairs or interacting groups (cf. West, 2012), synchronizing their states into either predominantly PEA or predominantly NEA states within an interacting subgroup. As this inter-correlation contagion occurs, local correlation in emotional states begins to dominate individual autonomy to a sufficient degree that its effects can no longer be ignored. That is, these local effects can no longer be assumed to cancel one another out as noise. Thus, these patterns constitute an emergent signal about a new property of the ecosystem. Specifically, the new property is as follows: If the emotional state of an individual is known, its state is predictive of the states of its neighbors, albeit probabilistically.

### Emotional States Enable Information Transit

When subpopulations within a broader population become inter-correlated, information about emotional states is transported between neighboring individuals. Through this social process, information about the ecosystem that is observed from different perspectives by a few individuals can be “stored” in the population’s organizing structure for retrieval and use by others. It is stored as ordering or symmetries within the correlated emotional states that “solidify” within the subpopulation. Because this signal would not exist (or would be very improbable) absent particularized disturbances in the environment, the stored information can be recognized and used by other individuals. This implies that information about the ecosystem that is stored in structure through emotional contagion can be observed and decoded by individuals to inform their behaviors even absent their own direct observation of events. Over time these regularities can become stable, reflecting a form of proto-organizing that enables efficacious action by a collective. This is akin to Prigogine (1995) nucleation mechanism in chemical systems.

The dynamic process of human proto-organizing is an example of emergence (Goldstein, 2011). It begins when information about the ecosystem “containing” the population of human beings is incorporated into population-level structures that govern individual-level interactions within the population. Information about the ecosystem is “sensed” by some (often at the periphery) who change their emotional state in response to a signal that they recognize—perhaps an opportunity for or risk to the population with an indeterminate (or probabilistic) flow-through impact on any given individual. For others inside the system with no direct knowledge of the original signal, the reason for a change in one’s emotional state is more unconscious than conscious and may also be influenced by relative status. As changes in the emotional states of others are observed or experienced, additional individuals synchronize their states. These changes begin to impact the state of the population *as a system* as a correlated state comes to span regions of the population comprising the system^1^. The synchronization may be conscious but it is more likely unconscious, like some biological imperative.

The process of emotional contagion is therefore fundamentally about information transit: information about opportunities or risks flows through the population, albeit without words for the most part, but rather mood. Note that the use of a previously developed symbolic language is not a prerequisite for this contagion. Although information is being transferred from person to person, this is not accomplished through symbolic language *per se*. A “system” of interacting individuals “senses” a disturbance in the ecosystem—an opportunity or a risk—and coordinates a synchronized response through emotional contagion alone in a manner analogous to neural networks (Hazy, 2012). In effect, organizing into groups, organizations, or institutions is enabled when the emotion state of an individual in the population can be predicted in part by the state of other individuals in its “domain” within the ecosystem. The population itself can be said to exhibit an observable emotional state, either leaning PEA or leaning NEA, and this aggregate state can be observed by randomly observing a large enough sample of individuals in the substrate population. One might observe this with a surrogate metric such as the consumer confidence index^®^, and the index value can vary by region, location or according to many other demographic factors. This can also be seen in the phenomenon of swarming in organizational or community change, during national change like in the Pax Romano or Arab Spring, or the US or French rebellions in the 1770s.

In the next section we describe parameters that describe the conditions that might lead to this capacity to recognize and respond to an external event in the ecosystem. Specifically in the model we are proposing, we adapt the model of Goldstein et al. (2010a,b) to argue that the enabling constraints in the ecosystem are represented by two control parameters, one regarding the physical event or disturbance, and the second involving the efficacy of emotional and informational exchange within the population itself. Each control parameter might include a threshold value after which a phase transition would unfold from a random distribution to the dominance of one state over the other or from one state to the other within the population.

## Parameterizing Emotional Contagion

This section proposes that emotional states and how they spread through the population are an observable biological mechanism that reflects conditions in the environment. In particular, this paper posits that the *potential* for inter-correlated emotional states within a population can be reflected as an *order parameter* that depends upon the interaction of two independent *control parameters*, each of which reflects the presence of relevant constrains within the ecosystem that are acting upon a population at a particular point. The two control parameters we propose are:

(i) the presence of a significant disturbance in the external resource flows that may have complex impacts on individuals or groups who are at that point in the ecosystem, and
(ii) the fidelity and complexity of the transit network for information about the disturbance that flows internally among individuals in the population.

This latter parameter relates to the density of social networks and the longevity of connections (and thus their trustworthiness) within the population. It might also relate to other factors such as ethnic and cultural partitions in society.

Following the model of social innovation of Goldstein et al. (2010a,b), threshold values serve as bifurcation or tipping points which might signal a possible phase transition, for example, between a predominantly NEA versus a predominantly PEA state in the population. We propose that each of these threshold values relates to one of the above external factors and is represented by an independent parameter. These are introduced here and then described in more detail in the next two sections.

**FIGURE 1:**
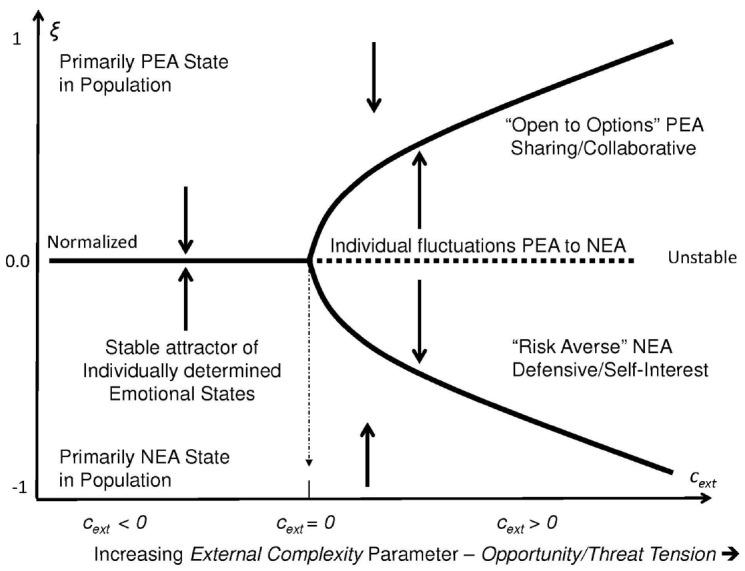
**As external complexity increases, the value of the order parameter ξ undergoes a *pitchfork bifurcation* that reflects stable levels of emotional states within the population.** When *c*_ext_ < 0 social pressure to conform emotionally is minimal and individuals respond independently with autonomy. The mean of the population is relatively stable. However as external complexity increases, a singularity or bifurcation point occurs at *c*_ext_ = 0 as individuals experience anxiety when they realize that their emotional response depends upon how others in the population react as well as what they themselves do. As *c*_ext_ > 0 increases the population moves into one of two synchronized dominate states each of which is stable: One is characterized as cooperating with others (under the PEA-dominant state) or competing with them (under the NEA-dominant state).

The first parameter measures *opportunity/risk tension*. It reflects the increasing external complexity, *c*_ext_, with regards to interpreting the relevance of disturbances in the ecosystem. Events in the environment are potentially relevant, but due to their complexity exactly how this might be so might not be clear to any particular individual. In these cases, addition information gathered from other individuals that might be used to triangulate the observations could be useful when attempting to decode information in the event. As shown in Figure 1, this parameter includes a threshold point, *c*_ext_ = 0, beyond which a physical disturbance in the ecosystem is recognized in the aggregate as potentially relevant to the population but the situation is sufficiently complex such that its relevance is ambiguous. In these cases, there is bi-stability in the emotional state of the population which is reflected as two possible stable levels of aggregate emotional response. The emotional state of the collective might fluctuate *en masse* from predominantly NEA to predominantly PEA and then back to NEA, depending on the flow of emotional state information within the population.

**FIGURE 2:**
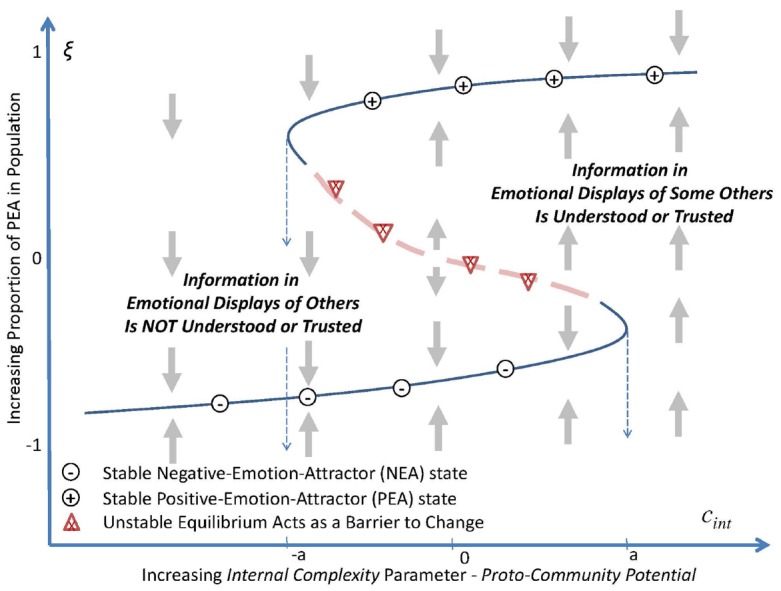
**When conditions are such that external complexity is greater than its bifurcation point, that is, *c*_ext_ > 0, as internal complexity, *c*_int_, increases (decreases) such that *proto-community potential* increases (decreases) to above a minimal (below a maximal) threshold, –*a* (or in the maximal case *a*), the order parameter passes through a *fold bifurcation* (also called a “tipping point”).** At points between –*a* and *a*, the order parameter exhibits bi-stability at either one of two stable levels of inter-correlated emotional synchronization within the population. For values of proto-community potential below –*a*, and above *a*, there is a single stable collective emotional state.

The second parameter *proto-community potential* measures the internal complexity, *c*_int_, and fidelity with which information and emotion spreads through a subpopulation. The potential of the information is reflected as displays of the emotional state that are recognized or “gathered,” by one individual about the emotional state (as a PEA or NEA) of an influential “other.” It describes the potential that the information will be (1) encoded, (2) shared as an emotional display or directly experienced, (3) recognized and trusted by others, and (4) “used emotionally” to adapt one’s own emotional state to synchronize with those with whom the individual interacts^2^ (cf. Aviezer et al., 2012). As shown in Figure 2, this parameter has two threshold values of internal complexity, *c*_int_ = *–a* and *c*_int_ = *a*, between which there are two stable levels for the aggregate emotional state reflected in the population.

### Opportunity/Risk Tension—Parameterizing the Reliability of Resource Environment

The first parameter, *opportunity/risk tension*, or *c*_ext_, measures the clarity with which signals about resource opportunities or risks present themselves in the ecosystem. As such, it reflects the level to which access to various resource reservoirs or sources of risk at a particular point in the ecosystem are unambiguously recognized to be changing in ways that are relevant to individuals and groups.

This parameter reflects how the changing potential for acquiring and using needed resources impacts the interactions among individuals in the population when they are treated as autonomous actors. When a potential opportunity or risk presents itself, each individual implicitly asks in the context of an emotional reaction: Do I (or we) prosper? Do I (or we) compete? Must I (or we) cooperate to maintain access to the resources? These questions create cognitive and emotional tensions among individuals struggling to answer them. As an example, consider how groups cooperate to build, maintain and defend a dam and reservoir in order to maintain a constant water supply in support of a safe community. Biologists who argue for group evolutionary selection processes call these collective dynamics “nesting safety” in support of a “defensible nest” (Nowak et al., 2010). The opportunity/risk tension parameter reflects the level of tension within an aggregate of the population that is due to environmental conditions in the ecosystem that might impact, either positively or negatively, the potential for nesting safety.

A simple (low complexity) event might be an unambiguous disturbance such as a fire in the theater. The appropriate NEA versus PEA is immediately clear to others when someone yells, “FIRE!” People do not spend much energy looking to others to confirm the danger. They immediately adopt an emotional state (most probably NEA) and take action to protect themselves and their dependent loved ones. Because this is a simple, unambiguous signal that does not require collaboration to decide on which action to take, except perhaps locally among families, an emotional state is adopted quickly with a high probability and with a single emotional interaction, or “dose.” Because the threat is apparent and clear and although there might be fear and anxiety, there is little internal tension; the transition to a dominant emotional state is continuous, and in this example, probably quite fast. In this simple case we say the value of the opportunity/risk tension parameter is less than 0, that is, *c*_ext_ < 0.

On the other hand, a disturbance might be less transparent and be difficult to interpret, for example changing weather patterns. In such cases, disturbances in the environment might be reflected as internal emotional disturbances within the community such as heated discussions and disagreements and the resulting emotional tension among those affected. Rituals like screaming matches, stylized displays of power, appeals to superstition, metaphoric “rain dances” or sacrifices might be used to evoke desired emotional reactions in others in an effort to synchronize emotional states.

A powerful competitor’s aggressive move into one’s market might require a response, but the specific response might be unclear. Does the organization abandon its market and move to a different one, cordon off a niche market and prepare to defend it, or to take some strong competitive initiative (perhaps legal action) to preserve access to its markets or other resources? The attack is a disturbance which causes an emotional response within the organization. However, in this case, the need to formulate a coordinated collective reaction creates internal tension in the organization as the collective’s emotional response only gradually becomes synchronized (more slowly than in the theater fire example). This class of disturbance requires multiple “doses” of emotional interaction to synchronize with others. As a result, there is usually a time delay. Under these complex conditions the opportunity/risk tension parameter is greater than 0, that is, *c*_ext_ > 0.

There is a point along this opportunity/risk continuum between simple conditions and more complex conditions is called the bifurcation point. This point distinguishes between conditions where disturbances are so transparent that individuals easily assume an emotional state with a single event or “dose” and those situations where disturbances are ambiguous, potentially impacting both the individual and the larger collective upon which each person depends, perhaps in complex ways. In this latter case, the individual seeks interactions with others to determine how one should respond. The bifurcation point is where *c*_ext_ = 0 (see Figure 1).

We argue that proto-organizing begins beyond the bifurcations point, where *c*_ext_ ≥ 0, when individuals must interact emotionally to know how they feel about events. It can be observed as repeated purposeful interactions within a subpopulation as individuals seek to stabilize their own emotional states and those of others through interaction. We call these ritualized initiations into a shared identity the community’s *priming rituals*.

In the aggregate, the opportunity/risk tension parameter measures how an aggregate in the population reacts to resource and risk conditions within its ecosystem. Using the venture capital ecosystem as an example, the opportunity/risk tension parameter might measure the flow of funds from limited partners into various types of venture funds and the flow of these funds into ventures at various stages of development. The availability of seed or early stage funding opportunities versus late stage financing might create different levels of tension among venture capital funding transactions. In cases where opportunities require small investments relative to the size of the fund and opportunities are ubiquitous, like SmartPhone applications soon after the Apple iPhone was introduced, seed and start-up funding is likewise ubiquitous. A single investor or perhaps an angel investor might quickly become excited into a PEA state and independently fund the venture. This would be cases where *c*_ext_ < 0.

More often, however, are cases where *c*_ext_ > 0. In these cases, investments are syndicated among several VC firms so that a group of individuals collectively becomes excited about the possibilities (or concerned about the risks) and together develop correlated PEA states (or NEA states). It is not surprising that these two markets have become increasingly differentiated since the internet bubble burst in 2000 and 2001 with individual angel investors focusing on very risky seed funding and professional venture capitalist syndicating less risky, later stage investing.

A fecund ecosystem can also be marked by competition. In these cases, events can signal a level *relative risk* rather than an unambiguous opportunity. The launch of the iPad created a substitution risk for PC makers such as Dell or HP, and even PC chip manufacturers like Intel or AMD. There are conditions therefore where the ecosystem presents opportunities and risks ambiguously. Small groups might form competing coalitions which creates tension—like those formed by contestants on the US reality TV show *Survivor*.

When there is little ambiguity along the opportunity/risk dimension, when *c*_ext_ < 0, one assumes that fluctuations between PEA and NEA occur autonomously in individuals across the population. Each individual independently stabilizes on a certain emotional state even from a single encounter with an environmental disturbance. They are either open to opportunity (PEA) or concerned about the risks (NEA). Under these conditions the emotional state in the population stabilizes at a certain level quickly and without much chatter. This stability represents what amounts to a proto-decision, a “gut check” about how one feels about the situation. As a result, when *c*_ext_ < 0 one would assume that for any random sampling of emotional states at a point in the ecosystem, on average there would be consistency about how many individuals are in one state or the other.

As the opportunity/risk tension parameter increases beyond the bifurcation point, that is where *c*_ext_ > 0, however, individuals are sensitive to the emotional tension of those around them. Emotional interactions ensue such that individuals stabilize at one or the other emotional state based upon not only their own encounter with news of the disturbance, but also in synchrony with the states of those with whom they interact. In this case, emotional contagion processes are involved and the resulting dominant mood can be positive, or it can be negative, but it is not mixed. It can also shift quite abruptly *en masse* from one state to the other, a condition of bi-stability. These different states are not independently distributed across the population. They are “clumpy” as emerging patterns can be observed in the emotional states of individuals across the population. These ideas imply the following propositions:

***Proposition 1A:*** *A parameter—called the **opportunity/risk tension** parameter—can be identified which reflects the transparency of disturbances in the ecosystem as well as how they are perceived emotionally by individuals and the speed with which emotional contagion might unfold in response to a disturbance in the environment.*

***Proposition 1B:*** *When a threshold value of this parameter is crossed, the emotional states of individuals are increasingly influenced by the emotional states of others (rather than their own independent reaction) and this creates the potential for bistability with two stable levels for the aggregate emotional state of the population.*

***Proposition 1C:*** *Crossing the threshold is signaled by priming rituals which indicate that proto-organizing has begun in an effort to locally synchronize emotional states.*

If one assumes 0 fidelity in the information transfer about emotional states among individual agents (i.e., they are members of what might be called different clans or enemy tribes), then individuals experience and exert no mutual influence. In these cases, knowing the state of one individual does not directly predict that of its neighbors, and there is no local correlation (other than close-in groups like families) except with regards the central tendency statistics^3^. The impact of “emotional connectedness” in the above proposition and the fidelity of information transit about emotional states as well as how these might lead to the formation of a proto-community identity are considered next in the context of a second parameter.

### Proto-Community Potential—Parameterizing Reliability of Socio-Emotional Environment

The second parameter, *potential for proto-community*, or *c*_int_, measures the complexity and reliability of the information from emotion that is available to individuals from the emotional displays of others in the subpopulation. This might depend upon the longevity and quality of trusting relationships connecting the individual with others. These relationships might be affected by the severity of predation or selection pressure in the ecosystem or competition among different community-identity groups (e.g., financial market or general business conditions). It might also be impacted by the level of competition versus cooperation within the broader population, and how these forces combine to cull the population eliminating some actors and thus weakening relationships and undermining trust. It also depends upon the capacity of individuals to recognize and imitate the emotional state of others (Aviezer et al., 2012).

This parameter reflects the extent to which the informational differences about emotional states present in the ecosystem transmit relevant signals accurately (with fidelity) throughout the subpopulations as they form proto-community identity. For example, is a warning signal that is issued by a particular individual trusted and heeded by others or is it ignored as a “boy crying wolf” as in the fairy tale? Does that individual’s emotional state spread to others and if so at what “infection” rate? Fluctuations like these can be seen in financial markets where information about exogenous macro-events is interpreted by traders within the markets and perceived opportunities or risks spread through the population of professional and then amateur investors through contagion. The density and connectedness, especially trusted connectedness of networks, are factors influencing this parameter. In business organizations, a possible surrogate metric at the firm level-of-analysis might be what Cohen and Levinthal (1990) call absorptive capacity which attempts to capture the ability of an organization to gather information from external events and then process it for use within its internal structure to enable efficacious organizing.

The emergence of a proto-community through these dynamics is evident in most sustainable community development efforts and missing in those that do not survive (Uphoff et al., 1998). In these cases, the initiators of the change process used village or neighborhood meetings with full adult participation in decisions to involve positive contagion with high fidelity and as a result a new community-identity emerged.

The value of this parameter reflects the level to which there is an opportunity for the individual to “synchronize” his or her emotional state with that of others who share a community-identity. By doing so, individuals can align emotionally to take advantage of an opportunity to gain resources or escape a threat that might impact the community. We argue that this “proto-organizing” only occurs in the interval between two extreme cases. For the case where the social network is sparse and weak with little trust and affinity, where *c*_int_ < –*a*, there is little opportunity for proto-organizing. In contrast, in the extreme case of very high proto-community with potential beyond a threshold value, *c*_int_ > *a*, there is very strong interconnectedness as one might observe within a nuclear family or a tightly integrated military unit. In these cases, the only stable emotional configuration would be the “we” or shared PEA state. In these highly integrated cases one might observe willingness for individuals to sacrifice themselves for their clan or unit.

Self-sacrifice in the case of high proto-community potential is in contrast to situations where the presence of informational differences among emotional states is closely held and not shared with others due to competition, or cases where signaling might actually be deceptive (e.g., as feigned madness, misdirection, or outright lying) to gain advantage in the struggle to position oneself to acquire and maintain access to resources and minimize risk. This latter case implies a possible imperative to “defect” in game theory terms and to not synchronize with others, influenced perhaps by a sense that the emotional information within the ecosystem is not reliable (Kidd et al., 2013). Between the threshold values, proto-organizing can occur.

To summarize, the proto-community potential parameter represents the potency of information gathering and use (Gell-Mann, 2002) for individuals. It measures how reliably the emotions of others can be used to determine an appropriate internal emotional and cognitive state in a distributed ecosystem. It measures the level to which individuals are able to use their social networks to identify and interpret distributed events and differences in individual perspective or experience as they assume an emotional state.

This article posits that the reliability of the transit of emotional state information reflects several elements of the underlying or substrate social network, including for example the density and trustworthiness of connections within the population (see Figure 2). For example, the perceived reliability of social connections could be affected by the severity with which evolutionary selection pressures (for example, layoffs, or turnover in business) have culled the population thereby eliminating trusted relationships (i.e., the iterated weeding out of trusted colleagues or the addition of strangers with unknown motives). This might tend to call into question the reliability of the information observed in the emotional states of others within a population. The less severe is the culling, the better understood and more trusted are the emotional pathways. Thus, under these conditions there is greater potential for efficacious collective emotional synchrony; the “infection” rate might be faster. These factors are indexed by the *proto-community potential* parameter as described in the following propositions:

***Proposition 2A:*** *A second parameter can be identified—called the* ***proto-community potential*** *parameter—as a metric reflecting the emotional connectedness characteristics of a given population.*

***Proposition 2B:*** *As this parameter increases, the potential for and speed of contagion also increases.*

***Proposition 3C:*** *As the parameter increases, a first threshold is crossed wherein one can observe the initiation of priming rituals that promote proto-organizing.*

***Parameter 2D:*** *As the parameter continues to increase, a second threshold is crossed which involves the disappearance of the self-interested socio-emotional state NEA state as a stable alternative; this transition is signaled by a willingness to sacrifice oneself for the greater good.*

### An Order Parameter for Proto-Community Formation

This section proposes an *order parameter*, ξ, which reflects an intensive property of a social system at work within an aggregate of individuals. Perhaps the relevant system is a group of individuals, an organization, or a firm. The value of the parameter is meant to reflect the presence of recognizable ordering among individuals who are being considered as an aggregate and who act as a system at a position in the ecosystem. The observed “ordering” might be a pattern or symmetry in a sub-population that signals the presence of “proto-organizing” within the aggregate. Because it is by definition relative, “ordering” is not detectable in any individual when viewed in isolation, and because it is not observable in components or elements (that is, individuals) of the system, one might say that it is an “emergent property” of the aggregate acting as a system (Goldstein et al. (2010a)).

An order parameter that reflects the presence of proto-organizing is the final factor needed to specify a mathematical relationship that might be used to describe the potential for individuals to form into a system of proto-organizing in the aggregate at a point in an ecosystem. A group of previously autonomous individuals might be organizing into a meeting, for example, and this might be observed as the stabilization of the order parameter. More specifically, under certain parametric conditions, the order parameter would identify the potential for discontinuous phase transitions between two dynamically stable emotional attractor states within aggregates of individuals in a population: One state would be ordered but stable in a certain way, positive and cooperative, for example. The other might be ordered and stable in a different way, negative and combative, in this example. The dominant emotional state of a meeting (that is, the aggregate is the set of participants in a meeting) that is being observed, could potentially change quite suddenly between states, for example, a property that cannot be reduced to the individual level.

The transition from one state or “phase” to another is thus measured by the order parameter. We define this order parameter, ξ, such that it is measured as points along the interval [–1, 1], with –1 reflecting a population with a 100% in one phase orientation, the NEA, and the value of 1 reflecting a population with a 100% in the other phase orientation, the PEA. Under this definition, a phase transition occurs when the order parameter changes from nearly –1 to nearly 1 or from nearly 1 to nearly –1 and remains dynamically stable for a time.

We propose that the potential for an aggregate to proto-organize can be measured by this order parameter. Further, we argue that the value of this potential, ξ, can be modeled as dependent upon the interacting values of the two control parameters discussed previously. The opportunity/risk tension parameter, *c*_ext_, reflects the ambiguity of opportunities or threats that might impact continuing access to resources in the environment (i.e., the reliability of the resource environment) for the aggregate. The proto-community potential parameter, *c*_int_, reflects the level of emotional communication, reputations, and trust among potential cooperating participants (i.e., the reliability of the socio-emotional information environment) within the aggregate.

To recognize a phase transition, we would be looking for an order parameter that measures the proportion of the population that vectors toward cooperative action, which we posit is the PEA state in the context of a complex and thus initially ambiguous resource environment (i.e., the “we” or “one” state, as relates to a specific shared identity) versus the proportion of the population that vectors toward defensive self-protective action, which we posit is the NEA state (i.e., the self-interested “I” or “me” state). This proportion would be measured as points along the interval [–1, 1], with –1 reflecting a 100% “me” orientation (individualized activity posited to be the NEA state), and 1 reflecting a population with a 100% “we” orientation (cooperative activity posited to be the PEA state). The order parameter ξ does exactly this.

Under this definition, a phase transition occurs with respect to individual alignment with regards a specific condition when the order parameter changes from a stable “me” dominated aggregate state to a stable “we” dominated aggregate state, or from “we” to “me” in a particular context. When there is opportunity/risk tension beyond the bifurcation threshold as shown in Figure 1, the unstable equilibrium value between these two extremes (which has been assumed to be normalized to 0 in this simplified model) represents a dynamic cross-over point. Above this point, individual emotional states are attracted to the +1 state of action orientation; below it they are attracted to the –1 state through emotional contagion processes.

Thus, we define the *order parameter*, ξ *ϵ* [–1, 1], to be a measurable quantity that can be normalized to values ranging from ξ = –1 when it is ordered in a shared NEA state, ξ = 0 when the system is in a mixed or independent and identically distributed (iid), and ξ = 1 when it is ordered in a shared PEA. This order parameter reflects, for a particular situation, the proportion of the population that buys-in to a particular shared emotional state, either one that favors cooperation (assumed to be PEA) or one that favors self-interest (assumed to be NEA).

***Proposition 3:*** *For a well specified condition impacting an aggregate in the ecosystem, the potential for a phase transition (or sudden discontinuous shift between points of bi-stability) in the order parameter* ξ *can be described as a function of the values of the* ***opportunity/risk tension****, c_ext_, and the* ***proto-community potential****, c_int_, parameters.*

Later, we will show that this relationship is the cusp of change model described by Goldstein et al. (2010a).

It may be the case that proto-organizing into a stable PEA state of an aggregate within a population is itself recognized by individuals as an event with potential implications for the individual. Recognizing this type of event can itself elicit a PEA or an NEA state in individuals. It is reasonable to assume that a positive feedback process might ensue such that additional individuals synchronize with the stable PEA state. What we are calling a *proto-community* is defined to occur when the chosen order parameter remains stable as some individuals leave the population aggregate and other new individuals join it and by doing so become socialized into the stable proto-organizing dynamic over time, perhaps through institutionalized priming rituals. Each newly joining individual either quickly synchronizes with the dominant PEA state and seeks to cooperate rather than going it alone, or chooses to leave the emerging proto-community, rejecting the socialization process.

Under these conditions, the proto-community’s shared PEA state supports the norm of cooperation with others who share the same community-identity through a reinforcing feedback loop as follows. When an individual’s community-identity is activated by events or conditions in the ecosystem, that individual’s PEA emotional state is likewise activated. This activation influences others in the community to synchronize their emotional state, and when they assume the PEA state, the first individual is positively reinforced in the PEA state, and so on, in an activation process much like a neural network (Hazy, 2012). The internal influence dynamics that enable and mitigate contagion are discussed next.

## A General Model of Contagion in Populations

Certain conditions can cause one or the other of these two emotional states, PEA and NEA, to be “contagious,” like an infection that spreads through the population. There are conditions and times when there is a disturbance in the environment that is detected by some individuals but potentially not others (like Obi-Wan Kenobi or Yoda sensing a disturbance in the force), some sort of opportunity or risk that is in excess of a measurable threshold, whereby the population takes notice, such as a coming financial crisis or a shortage in the oil or water reserves^4^. A sudden change in the dynamic stability of the aggregate measure of a population’s emotional state in response to a disturbance in the environment would signal a possible phase transition relevant to the group, organization, or community.

The challenge for research is to identify and measure the conditions in the ecosystem under which the population is stirred through emotional contagion to respond *as a system*. That is, under what conditions would the emotional states of individuals become synchronized into a coherent response of individuals in a population acting together as a system. For modeling purposes, “acting together” is measured by an *order parameter* as described in the last section.

To illustrate this idea, emotional contagion and its relationship to coordinated action could be observed in the response of financial policy makers around the world after the fall of Lehman Brothers in September 2008 (Investopedia, 2009). Swarming behavior in response to challenges to the population can be observed in social animals as well as human beings (Camazine et al., 2001). Here we explore the drivers that determine individual states during local interactions, the distribution of these states within the population, and how and with what parameters can one model the process of emotional contagion.

### Modeling the Dynamics of Emotional Contagion

As we describe in this and the next main section, emotional contagion occurs when a person in either the PEA or NEA state begins influencing others to synchronize with them into the same state. This transfer of state through interaction is referred to as the transit of information (about their respective emotional state). Physiologically, through the mechanisms of mirror neuron networks (Iacoboni, 2009) and hemodynamic sympathetic networks (Decety and Michalska, 2010), one person can stimulate the same emotional state in another within milliseconds (keeping in mind the above factors).

As a result of the transit of information, the activation of a synchronized emotional state within a population can occur unconsciously and quickly, and thus a common state can potentially spread across the entire population. A sudden change in the aggregate state—as measured for example by the normalized consumer confidence index^®^—is called a *phase transition* within the population (West, 2012). Of interest in this article are the parametric conditions in the ecosystem—that are both exogenous to and endogenous within an aggregate or sub-population—interacting to trigger a phase transition within the population and thus enabling proto-organizing.

In particular, as is described in the next main section (also see Dodds and Watts, 2004), the flow of information, including emotional states, across the population can vary depending upon the several conditions that reflect the strength of community ties. One important factor would be the probability that a given interaction will result in the transfer of relevant information from the informed party to the ignorant party. Does the informed individual even bother to warn the uninformed one by either hiding or displaying one’s emotional reaction? Is the second individual even “dosed” with the “infection”?

A second factor would relate to the strength of the warning offered during the interaction. A subtle, ambiguous, or indirect emotional expression related to a resource opportunity or risk is quite different than an impassioned argument in favor of community-level mobilization to action among connected others. In infectious disease term, what is the dosage level?

Beyond this, a third factor is how broadly and consistently the story is told. Does the informed party treat everyone the same, with the same impassioned warning, or is the emotional display more selective? Does one only feel free to display emotion openly in a close-in group, or more broadly? What is the distribution of dosage size? Finally, the uninformed must recognize the message (that is, an emotional display indicating fear, for example) and react, changing state to synchronize with the informed voice. How do people react? Does everyone react the same way? Are they different? What is the distribution in the population? All of these questions must be explored when modeling proto-organizing and the conditions which engender the formation of proto-community identities.

### The Dodds and Watts General Model

To explore this question, we will examine the generalized model of contagion developed by Dodds and Watts (2004) from standard epidemiology models. For our purpose, we will say that the “infection” they describe is a PEA state, although it could just as well be the NEA state that becomes infectious. The Dodds and Watts (2004) model is defined as follows. A population of N individuals is divided into three subpopulations of individuals with three different states, *S* (susceptible), *I* (infected) or *R* (removed). Note that *I* + *S* + *R* = *N*, [and in our case, *I* is the proportion of the population with a PEA state]. At each time step, *t*, each individual *i* *ϵ* N comes into contact with another individual *j* *ϵ* N which is randomly selected from the population. Infection occurs as follows. If *i* is susceptible and *j* is infected [with PEA], then *i* receives a dose *d_i_*(*t*) (of positive emotion) with probability *p* drawn from a distribution *p* *ϵ* *f*(*d*). If *i* is not susceptible or is removed, *d_i_*(*t*) = 0. Each individual maintains a memory (i.e., neurological or psychological memory) of doses received over the last T time steps, remembering a cumulative dose of *D_i_(t)* = $\Sigma_{t^{\prime }=t-t+1}^{t}d_{i}\left(t^{\prime }\right)$. Individuals become infected when *D_i_*(*t*) > d_i_*, *i*’s dose threshold. Each d_i_* is drawn randomly at time *t*_0_ from the distribution *g*(*d*). This means that each individual can take only so many “doses” of positive emotion from others during an interval of time (which reflects memory of prior events) before he or she is “infected” by a PEA, state. Further, this threshold might vary among individuals.

Dodds and Watts (2004) continue their model to include situations where individuals recover from the “infection” (and are “Removed” from the infected population) if their cumulative dosage falls below their threshold. They also might become at risk for reinfection (perhaps with a new threshold) if they interact with an infected individual again. If the dosing (of positive emotion) that an individual is receiving from others drops below a threshold, then the person will either return to the more defensive NEA or stay in the PEA but at a low level of intensity. They point out that these dynamics can be quite complex, but for the simplified case, where the rate of removal, *r* = 1, and the rate of becoming re-susceptible ρ = 1, the system can be solved analytically. This means that falling below the infection threshold returns the individual to the susceptible population so that *R* = 0 and the population *N* = *I* + *S*. [For our purposes, *N* = *I* + *S* is the number of individuals, *I*, who are a PEA state, plus *S*, the number in a NEA state.]

To inform our discussion, it is useful to point out that Dodds and Watts (2004) report a series of interesting results from their model even for this simplified case. In particular, they show that the contagion dynamics within a population can vary considerably depending upon several exogenously determined conditions such as: the probability of receiving a positive dose, the distribution of dosage size, and the distribution of individual dose-size-thresholds. Varying these factors individually and in combination can be used to replicate many models of social contagion that have been used in other studies. Because we are interested in social contagion that results in shared emotional reactions to conditions in the ecosystem, it is important to note that the flexibility of this model derives from the introduction of individual memory and thus an accumulated dosage calculation that relates to an individual’s threshold of infection. Memory to store information about prior interactions enables many possible contagion relationships.

By comparing the probability *P*_1_ that an individual is infected on the first contact with the probability *P*_2_ that an individual will be infected on the second contact, and where T is the memory parameter that describes the time interval wherein dosage is accumulated for effect, Dodds and Watts (2004) divide the dynamic course of contagion into three classes^5^. Although the different classes identified by Dodds and Watts (2004) point to the richness of the possibilities for future research, herein we limit our discussion to just two cases.

The analysis of Dodds and Watts (2004) suggests circumstances where two distinct dynamic situations exist depending upon parametric conditions. On the one hand, if the probability that an individual will assume the emotional state of another with the first interaction, is greater than half the probability that it will assume it on the second interaction, we say that there is no relevance to further socio-emotional reinforcement and assume that these individuals offer little resistance to infection to emotional exchange and are likely to be infected upon first encounter.

On the other hand, if the probability of adopting the emotional state of the other on the first interaction *is less* than half the probability of adopting it after the second interaction, then there is an apparent need for socio-emotional reinforcement. This suggests that there is resistance to independent emotional influence. This latter case exhibits the conditions where opportunity/risk tension and proto-community potential interact to enable a discontinuous phase transition in emotional state. Under these conditions multiple emotional displays from others are needed before an individual synchronizes emotional state with others.

There are many empirical questions that are embedded in this argument. For example, the nature of the PEA state is more neurologically open to new ideas, and this may result in people who are in a PEA state being slightly more vulnerable to contagion. On the other hand, the defensive and ecologically imperative nature of the NEA as a basis for survival may result in people having a default condition of reverting to the NEA, rendering them more vulnerable to infection or contagion from others in the NEA state. The documented evidence claiming that negative emotions are stronger than positive (Baumeister et al., 2001), supports a non-random sensitivity to contagion of people who begin in the NEA versus PEA state.

## A Simple Model of Proto-Community Emergence

**FIGURE 3:**
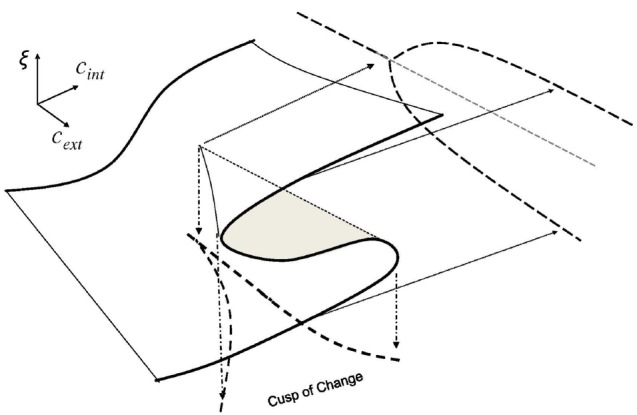
**The Cusp of Change Model depicts the parametric interaction—between the *Pitchfork Bifurcation Model* in the parameter *c*_ext_ from** Figure 1 **and the *Fold Bifurcation Model* (also called a “tipping point”) in the parameter *c*_int_ from** Figure 2**—that implies a cusp-shaped region of bi-stability.** Within this “cusp of change” region of proto-organizing potential, complex contagion dynamics are characterized by synchronized emotional states within the population as entire regions of the population exhibit correlated emotional states that can change to the other state *en masse*. The text describes why the cusp of change is where proto-organizing occurs.

In this section we develop a simple model of a phase transition in emotional states from a “me” orientation to a “we” orientation in human interaction dynamics (HID). To explore this dynamic, we build upon the cusp of change model described by Goldstein et al. (2010a,b) which uses the canonical mathematical model for one order parameter, ξ, and two control parameters, *c*_int_ and *c*_ext_. In our case, we define the parameters *c*_ext_ and *c*_int_ to be *opportunity/risk tension* and *proto-community potential* respectively. Together, these parameters describe the complex dynamics of proto-organizing. This is shown in Figure 3. Further, for *c*_ext_ > 0 and for values of *c*_int_ between the curves of the cusp, there are conditions (i.e., inside the cusp-shape) where the order parameter experiences bi-stability wherein the population has two stable conditions: either predominately PEA or predominantly NEA for that subpopulation.

### Emotional Contagion and the Cusp of Change Model

We assume that for a given subpopulation, emotional contagion occurs when the order parameter for a subpopulation changes significantly and becomes stable at a new level, an event that signals to observers that proto-organizing is occurring. The signal is observable because one can recognize that the locally synchronized emotional states within the subpopulation have become differentiated from the background and stabilize at that differentiated level. As this occurs, individual emotional states spread through the subpopulation as individuals form a shared community-identity of “us” and “we” that is rooted in emotional synchrony and establishes the subpopulation as persistently different than the background at least in this one aspect. This is a proto-community.

#### The Single Stable-State Case of Emotional Contagion

For simplicity, we initially assume from the Dodds and Watts (2004) model that the probability of infection from the first interaction *P*_1_ is high and (specifically that *P*_1_ ≥ *P*_2_/2 where *P*_2_ is a second interaction). This is the case when the opportunity/risk tension parameter *c*_ext_ < 0 which is outside the cusp of change in Figure 3. Dodds and Watts (2004) show that these conditions imply a deterministic threshold (DT) model of social contagion. In this case, as disturbances occur in the environment, synchronized emotional states spread by contagion through the entire population and are localized in dynamically stable subpopulations. In this case, no clumping would be observed and no proto-organizing would occur in subpopulations because no subgroups would deviate significantly from the changing substrate.

#### The Bi-Stability Case of Emotional Contagion Inside the Cusp

In cases identified by Dodds and Watts (2004) where *P*_1_ is lower (specifically that *P*_2_ > *P*_1_/2 ≥ 1/*T* where *P*_2_ is a second interaction and *T* is the length of memory) two stable states might be possible within various regions or subpopulations of the overall population. (From Figure 3 note that this is where the *opportunity/risk tension parameter c*_ext_ > 0 in the cusp of change model and where the *community-identity potential* parameter *c*_int_ is between two threshold or “tipping point” values.) In these cases, path-dependence affects tend to make emotional states “sticky” in these subpopulations as a means to sustain proto-organizing potentials.

This local dynamic implies that phase transitions in the broader population can occur if the HID are such that proto-communities in subpopulations also synchronize with one another across the entire population. These interaction dynamics are assumed to relate to social or emotional contagion processes internal to the population, and these may not be homogeneous. For these cases, Dodds and Watts (2004) work shows that the specific dynamics of contagion in the broader population can be quite complex. Thus, the exact nature of the course of the “infection” through the population will ultimately be explored via empirical studies and improved modeling techniques.

However, to advance this thinking initially, we propose the Cusp of Change Model (Goldstein et al., 2010a,b) with well-specified assumptions as a starting point—a complement to the general model of Dodds and Watts (2004). This model describes how emotional contagion can act as a mechanism that enables proto-organizing within subpopulations under certain parametric conditions. In addition, this model might also shed light on when proto-organizing remains stable as subpopulations change their constituency over time (as occurs in firms and other organizations). Such a model would describe the formation of proto-communities.

### The Mechanism of Emotional Contagion

To recapitulate, emotional contagion is the process where positive or negative emotional states spread through and synchronize within a population via emotional and social interaction. It can occur gradually in response to changing opportunity/risk conditions or it can be sudden, a phase transition. The latter case is enabled when two factors interact to create the requisite enabling conditions as shown in Figure 3. These conditions exist when:

(1) The parameter *opportunity/risk tension* in the ecosystem is such that threshold is crossed whereby the complexity of the situation is such that isolated individuals have difficulty responding emotionally absent interaction with others, and
(2) The parameter *proto-community potential* or clumping of proto-organizing within the population is in a range whereby it is complex enough that individuals identify with the emotional reactions of others sufficiently to use that emotional input as a partial determinate of their own reaction, but the complexity is also low enough that they do so with skepticism and do not immediately adopt the emotional state of another after a single interaction.

Under these parametric conditions, the process unfolds as follows: a subset of individuals who find themselves in a position to recognize the opportunity or risk directly in the environment assume varying emotional states, consciously or unconsciously. Through interactions with others, these emotional states can spread to others who did not directly observe the event. The likelihood of “infection” depends upon each individual’s susceptibility to socio-emotional influence from a particular interaction including relative status (Hazy, 2012). Local stability is enabled when local proto-organizing conditions support local synchronization and offer resistance to outside influences which would otherwise destabilize a proto-organizing subpopulation. Building upon the Dodds and Watts (2004) results, this implies:

***Proposition 4:*** *When opportunity/risk tension conditions in the environment would imply a emotional reaction to events is warranted, two individuals, called “ego” and “alter” interact emotionally during a finite time step, an “interaction.” The likelihood that an alter agent will synchronize with the ego (become infected with its emotional state) is positively related to:*

A. A. the level of emotional display by the ego, the “dosage,”
B. total dosage accumulated within the alter from recent prior interactions,
C. the number of recent interactions that are accumulated by the alter in “memory,” and
D. a level that exceeds the alter threshold beyond which an accumulated dosage implies synchronization, i.e., an infection, of emotional state.

In this way, even subtle and complex forms of organizing behavior can be modeled, providing powerful methods for better understanding human social and emotional organizing potentials and outcomes—the goal of HID as described by Hazy and Backström (2013). This also suggests:

***Proposition 5A:*** *When the* ***opportunity/risk tension*** *threshold is crossed and the* ***proto-community potential*** *parameter is in the bi-stable range between two tipping points, there are two stable levels of aggregate emotional states which constitute proto-organizing in a subpopulation.*

***Proposition 5B:*** *When these parameters imply that a subpopulation is in this “cusp of change” that subpopulation can switch between two stable emotional states due only to local internal proto-community dynamics that impact the factors described in Proposition 4.*

Note, however, that emotional contagion alone does not unite individuals into a cooperative effort. Rather it just gets people into similar emotional states, either open to influence from others (PEA) or suspicious of it (NEA). We also suspect that because negative emotions are stronger than positive ones (Baumeister et al., 2001), the NEA may be invoked with lower thresholds than the PEA. This also makes sense from an evolutionary perspective—it is more important for survival to be defensive in the presence of a risk or potential threat than to feel elated in response to an opportunity for good feeling.

## Concluding Observations

We have presented a theoretical and mathematical approach for describing the formation of proto-communities as a first step to social organization. This proto-organizing occurs through the process of emotional contagion. The paper includes propositions intended to guide future research.

The theory identifies the drivers and mechanisms that describe how synchronized emotional states emerge. This occurs through what amounts to a swarming process in insects and is related to infectious disease and social contagion dynamics that have long been studied and modeled in epidemiology. The point of departure here is the argument that human organizing itself is enabled by changing emotional states rather than rational choice.

Our premise is that when emotional states are synchronized through emotional contagion, a proto-organizing state emerges within the population, and this is the mechanism that enables coordinated action, including rational planning activities and the implementation of action plans. Further, the emergence of an emotionally enabled proto-organizing state precedes rational choice (perhaps only momentarily) and is a necessary precondition to the development of a rationally understood organizing structure.

Thus, this paper contributes not only new theory, but also a whole new perspective on organization theory. It proposes a framework which elevates the utility of emotional experience in organizations to an equal level with the rational-centric perspective that is usually the implicit orientation of management research. It makes the bold argument that organizing begins with and is enabled by emotional processes rather than rational ones. Emotions come first, and rational experience augments emotional experience rather than the other way around. The inverted perspective identified here, if supported empirically, would make it clear that the effective navigation of the emotional landscape in unfolding organizations is an essential skill for managers and leaders. It is central to success at all levels, and as such it deserves adequate focus. This is in contrast to the vast majority of the management literature where emotional experiences are considered an annoyance at best, are ignored as irrelevant most of the time, or in some cases they are even highlighted as a dangerous distraction to be avoided by skilled practitioners.

Recent advances in neuroscience, contagion modeling, and the complexity notion of swarming offer an opportunity to change this bias. They imply that it is time for organization and management research to better explore the organic nature of human organizing in ways that include both **emotional** and rational dynamics, and to learn from each and from both. In today’s flattening world and globalizing economy, it no longer makes sense to treat organizations as machines that are rationally designed by some and dispassionately submitted to by others, a sterile and unfriendly world where emotions have no place. Human beings organize emotionally. The tools to understand how this happens are available. It’s time to put them to work.

### Conflict of Interest Statement

The authors declare that the research was conducted in the absence of any commercial or financial relationships that could be construed as a potential conflict of interest.

## Footnotes

1 As proto-organizing structures unfold across the organization, influence is not homogeneous. Individuals vary in status for example (Hazy, 2012). An influence process emerges across the interactions *in the system* constraining the degrees-of-freedom of individuals as they interact at the fine-grained level.

2 Note that information about a disturbance in the ecosystem that is recognized by an individual might benefit others or it might be used against them. Deception in organizations might be an interesting area of research in the emotional contagion context.

3 For example the mean might be 50% PEA or it might be 70% PEA, and this would predict, but knowing what the neighbors are does not predict.

4 As pointed out by one of our reviewers, there is an important philosophic question here as to whether such changes can also be initiated endogenously, or whether there is always an external cause. This question is caught up in how one defines the system, of course. For this analysis, we take the relatively hard position that the cause is always external from the position of the system under study.

5 More specifically, in Class I, where *P*_1_ ≥ *P*_2_/2, the probability of infection on the first interaction is high enough such that the model implies behavior similar to *epidemic threshold models* also called *deterministic threshold models*. This means that as the probability that an interaction results in a positive dose increases, the steady-state percentage of infection at which the population stabilizes—called the fixed point attractor for a fixed value of the parameter—increases as the value of the parameter setting increases. Further, this relationship has a positive slope—as the parameter setting increases, the stabilizing point also increases. In Class II, where *P_2_* > *P*_1_/2 ≥ 1/*T*, the probability of infection after a second interaction is high enough relative to that from the first interaction and for a given length of memory that there are cases wherein a critical mass might be necessary to achieve a stable fixed point attractor level of infection within the population. This behavior is similar to *vanishing critical mass models*. In Class III, where 1/*T* > *P*_1_, the probability of infection on the first interaction is so small that a preexisting critical mass is always necessary if the infected population is to stabilize at a level greater than 0—whatever the probability that an interaction results in a positive dose of the infection—to imply a stable fixed point attractor level of infection. This means that the model implies behavior similar to *pure critical mass models*.
